# *Oryza sativa* BRASSINOSTEROID UPREGULATED1 LIKE1 Induces the Expression of a Gene Encoding a Small Leucine-Rich-Repeat Protein to Positively Regulate Lamina Inclination and Grain Size in Rice

**DOI:** 10.3389/fpls.2017.01253

**Published:** 2017-07-17

**Authors:** Seonghoe Jang, Hsing-Yi Li

**Affiliations:** ^1^Biotechnology Center in Southern Taiwan of Agricultural Biotechnology Research Center, Academia Sinica Tainan, Taiwan; ^2^Institute of Tropical Plant Science, National Cheng Kung University Tainan, Taiwan

**Keywords:** leaf inclination, grain size, plant architecture, transcriptional activator, transgenic rice, *Oryza sativa*

## Abstract

*Oryza sativa BRASSINOSTEROID UPREGULATED1 LIKE1* (*OsBUL1*) positively affects lamina inclination and grain size. *OsBUL1* knock-out (*osbul1*) plants as well as transgenic rice with reduced level of *OsBUL1* expression produce erect leaves and small grains. Here, we identified a putative downstream gene of *OsBUL1, OsBUL1 DOWNSTREAM GENE1* (*OsBDG1*) encoding a small protein with short leucine-rich-repeats by cDNA microarray analyses in the lamina joint and panicles of wild-type and *osbul1* plants. Transgenic rice plants with increased *OsBDG1* expression exhibit increased leaf angle and grain size, which is similar to an *OsBDG1* activation tagging line whereas double stranded RNA interference (dsRNAi) lines for *OsBDG1* knock-down generate erect leaves with smaller grains. Moreover, transgenic rice expressing *OsBDG1* under the control of *OsBUL1* promoter also shows enlarged leaf bending and grain size phenotypes. Two genes, *OsAP2* (*OsAPETALA2*) and *OsWRKY24* were identified as being upregulated transcriptional activators in the lamina joint of p*OsBUL1*:*OsBDG1* plants and induced expression of the two genes driven by *OsBUL1* promoter caused increased lamina inclination and grain size in rice. Thus, our work demonstrates that a series of genes showing expression cascades are involved in the promotion of cell elongation in lamina joints and functionally cause increased lamina inclination.

## Introduction

Crops are generally grown at high planting density in the field. For better yield and resistance against lodging, cereal crops, such as rice, with semi-dwarf and/or erect leaf phenotypes are strongly desired (Van Camp, 2005; Sakamoto et al., 2006). A rice leaf is composed of a leaf sheath, leaf blade, and a lamina joint (area between leaf blade and sheath, also called the collar) with auricles and ligule (Hoshikawa, 1989). In particular, leaf angle (the degree of bending between the leaf blade and leaf sheath) is one of the key agronomic traits that affects crop architecture and grain yields (Sinclair and Sheehy, 1999). Crops with erect leaves capture more light for photosynthesis and are also desirable for dense planting, all of which increase yields (Sakamoto et al., 2006).

Previously, it was shown that both phytohormone and non-hormone-related genes are involved in controlling the lamina inclination.

The brassinosteroid (BR)-deficient mutant *dwarf4-1, ebisu dwarf* (*d2*), and *dwarf1* (*brd1*), the BR signaling mutant *d61-7*, and plants with suppressed expression of *OsBRASSINAZOLE-RESISTANT 1* (*OsBZR1*) encoding a transcription factor involved in the BR signaling cascade display erect leaves (Hong et al., 2002, 2003; Sakamoto et al., 2006; Bai et al., 2007) while overexpression of BR biosynthesis genes or signaling components resulted in large leaf inclination (Yamamuro et al., 2000; Bai et al., 2007). For example, transgenic rice plants overexpressing sterol C-22 hydroxylase (a rate-limiting enzyme in BR biosynthesis) showed increased lamina angles (Wu et al., 2008). In addition to BR, other phytohormones are also involved in controlling the lamina inclination of rice. Ethylene participates in the response of BR-induced lamina joint inclination, and auxin (IAA) influences the lamina joint inclination at high concentrations and has a synergistic interaction with BR (Cao and Chen, 1995; Nakamura et al., 2009). Reduced expression of *SPINDLY*, a negative regulator of gibberellin (GA) signaling, also causes increased lamina inclination (Shimada et al., 2006). In addition, transgenic rice plants with overexpression of *LAX PANICLE (LAX)* (Komatsu et al., 2003), *OsILI-BINDING HLH1* (*OsIBH1*) (Zhang et al., 2009) and T-DNA insertion mutants of *OsWRKY11* (Wang et al., 2005), *OsLIGULELESS1* (*OsLG1*) encoding a *SQUAMOSA* promoter binding domain protein (Lee et al., 2007) exhibited erect leaves, whereas the double stranded RNA interference (dsRNAi) transgenic lines for rice MADS-box genes belonging to the SHORT VEGETATIVE PHASE (SVP) group such as O*sMADS22, OsMADS47*, and *OsMADS55* showed increased lamina angles (Lee et al., 2008). Decreased expression of *OsLIC* encoding a CCCH-type zinc-finger protein also results in increased lamina inclination through regulating BR signaling (Wang et al., 2008). It was also reported that *LC2* encoding a VERNALIZATION INSENSITIVE 3 (VIN3)-like protein acts as a repressor of cell division for regulation of collar development and the *lc2* mutation caused increased leaf angles (Zhao et al., 2010). A recent report showed that an activation tagging line of rice, *slender grain Dominant* (*slg-D*), with enhanced expression of a gene encoding BAHD acyltransferase-like protein, produced slender grains with enlarged leaf angles (Feng et al., 2016), and a gain-of-function epiallele of rice *RELATED TO ABSCISIC ACID INSENSITIVE3* (*ABI3*)*/VIVIPAROUS1* (*VP1*) 6 (*RAV6*) encoding a B3 DNA-binding domain-containing protein caused larger lamina inclination but smaller grain size by modulating BR homeostasis (Zhang et al., 2015). Moreover, induced expression of genes encoding atypical Helix-Loop-Helix (HLH) proteins such as *BRASSINISTEROID UPREGULATED1* (*BU1*; Tanaka et al., 2009), *Oryza sativa BU1 like 1* (*OsBUL1*; Jang et al., 2017), *INCREASED LAMININAR INCLINATION* (*ILI*; Zhang et al., 2009) and *POSITIVE REGULATOR OF GRAIN LENGTH 1* (*PGL1*; Heang and Sassa, 2012) or basic HLH (bHLH) proteins including *OsBUL1 Complex1* (*OsBC1*; Jang et al., 2017) conferred rice plants higher lamina angle degree with increased grain size.

In this work, transcriptomes in collars and panicles of *OsBUL1* null mutants and WT plants were analyzed and *OsBUL1 DOWNSTREAM GENE1* (*OsBDG1*) was identified as a putative downstream gene of *OsBUL1*. It encodes a small leucine rich repeat (LRR) protein possessing cell elongation activity. Sequentially, *OsAP2* and *OsWRKY24* are identified as putative downstream genes of *OsBDG1*, and functional activities are assessed with respect to rice lamina inclination and grain size. Both genes are able to increase lamina inclination and grain size under the control of *OsBUL1* promoter. We, therefore, provide a sequential flow of acting genes from *OsBUL1* for positive effects on rice lamina joint inclination and grain size likely through cell elongation.

## Materials and Methods

### Plant Materials and Growth Conditions

The activation tagging line of *OsBDG1*, PFG_1B_05536 is in Dongjin background (Jeong et al., 2006). Transgenic rice plants were produced with Tainung67 (TNG67) japonica rice cultivar. Rice plants (*Oryza sativa*) were grown in the field under natural long days or in the greenhouse with 28°C day/25°C night cycles. To assess the leaf angles of mature rice plants in the paddy field, leaves were numbered from top to bottom. Lamina angles were measured between a stem and a leaf blade at the second or the third leaf from eight to twelve individual plants per line when panicles come out of flag-leaf sheaths. Transgenic plants used for analyses in this work are all T3 or T4 independent lines.

### Lamina Joint Inclination Bioassay

Sterilized seeds were germinated and grown for 10 days in a dark chamber. Lamina joint inclination bioassays were performed as previously described (Jeong et al., 2007). Seedlings were sampled by excising approximately 2 cm segments that contained lamina joints at the same position from each plant under dim light condition. They were floated on distilled water containing various concentrations of BL. After incubation in a dark chamber at 28°C for 2 days, the angle induced between the lamina and the sheath was measured.

### Vector Construction and Transformation

Each open reading frame (ORF) of *OsBDG1, OsAP2*, and *OsWRKY24* was cloned into pGA3426 (Kim et al., 2009) or its derivatives for overexpression and/or dsRNAi purposes in rice. For expression by *OsBUL1* promoter, the ubiquitin promoter of pGA3426 was replaced with the 2.2 kb-*OsBUL1* promoter (Jang et al., 2017). Constructed plasmids were individually transformed into embryonic calli of TNG67 rice cultivars by *Agrobacterium*-LBA4404 mediation as described previously (Jeon et al., 2000).

### Hormone Treatment

Ten-day-old rice (*O*. *sativa* cv. TNG67) seedlings grown in the growth chamber were treated with brassinolide (1 μM, BL from Sigma–Aldrich) or GA (100 μM GA3 from Sigma–Aldrich). Whole parts above the roots were harvested for RNA extraction at 24 h time point after treatment.

### Total RNA Isolation and Quantitative RT-PCR Analysis

Total RNAs of all the materials harvested were isolated using RNeasy plant mini kit (Qiagen) or Trizol solution (Invitrogen) according to the manufacturer’s instructions. RNAs after DNase treatment were subjected to reverse transcriptase reactions using oligo(dT) primer and Superscript III reverse transcriptase (Invitrogen) according to the manufacturer’s protocol. Subsequent PCR was conducted with the first-strand cDNA mixture and EX-Taq polymerase (Takara Bio). Quantitative PCR (qPCR) was carried out by a CFX96TM real-time system (Bio-Rad) using Maxima SYBR Green qPCR Master Mix (Thermo). For PCR, each sample was analyzed in triplicate. The run protocol was: denaturation at 95°C for 10 min and annealing/extension repeated 45 times (95°C for 15 s and 60°C for 30 s, data acquisition was performed). Housekeeping genes such as *OsUBQ* (Komiya et al., 2008) and *OsAct* (Caldana et al., 2007) were included in the reactions as internal controls for normalizing the variations in the amount of cDNA used (Guénin et al., 2009). The threshold cycle (C_T_) was automatically determined for each reaction by the system set with default parameters. The specificity of the qRT-PCR was determined by curve analysis of the amplified products using the standard method installed in the system. Information on primers used is presented in **Supplementary Table S1**.

### Microarray Analyses

Collars of 80-day-old rice plants and young panicles less than 10 cm in length were harvested for RNA extraction for microarray. We used the Rice Whole Genome OneArray v1.1 (Phalanx Biotech Group, Taiwan) containing 22,003 DNA oligonucleotide probes. Each probe is a 60-mer designed in the sense direction. Among the probes, 21,179 probes corresponded to the annotated genes in the RGAP v.6.1 and BGI database. Additionally, we included 824 control probes. The detailed descriptions of the gene array list are available from http://www.phalanx.com.tw/products/RiOA_Probe.php. Fluorescent antisense RNA targets were prepared from 1 μg total RNA samples with the OneArray Amino Allyl aRNA Amplification Kit (Phalanx Biotech Group, Taiwan) and Cy5 dyes (Amersham Pharmacia, United States). Fluorescent targets were hybridized to the Rice OneArray with Phalanx hybridization buffer using Phalanx Hybridization System. After hybridization for 16 h at 50°C, non-specific binding targets were washed away by three washing steps (Washing I, 42°C 5 min; Washing II, 42°C, 5 min; 25°C, 5 min; Washing III, rinse 20 times), and the slides were dried by centrifugation and scanned by an Agilent G2505C scanner (Agilent Technologies, United States). The Cy5 fluorescent intensities of each spot were analyzed by GenePix 4.1 software (Molecular Devices). The signal intensity of each spot was introduced into Rosetta Resolver System (Rosetta Bio-software) to process data analysis. The error model of the Rosetta Resolver System could remove both systematic and random errors from the data. We filtered out the spots whose flag was less than 0. Spots that passed the criteria were normalized by 50% media scaling normalization method. The technical repeat data was tested by Pearson correlation coefficient calculation to check the reproducibility (*R*-value > 0.975). Normalized spot intensities were transformed to gene expression log_2_ ratios between the control and treatment groups. The interest spots which show significant differences were selected by log_2_ ratio ≥ 1 or log_2_ ratio ≤-1 and *P* < 0.05. Two independent biological replicates of hybridizations were performed.

### Histological Analyses

Lamina joint samples were fixed by 4% paraformaldehyde in 0.1 M sodium phosphate buffer, dehydrated through a series of graded ethanol baths, replaced with xylene, and embedded in Paraplast plus (Sigma–Aldrich). Paraffin sections (12 μm) were cut and stained with filtered 1% toluidine blue. The sections were photographed under a light microscope (Olympus BX51).

### Subcellular Localization of Proteins

For cellular localization of OsBC1, OsBDG1, OsBUL1, OsAP2.2, and OsWRKY24 in rice, either yellow florescence protein (YFP):gateway (GW) or cyan fluorescent protein (CFP):GW vector was used for the florescence fusion as described previously (Jang et al., 2015). Isolation and transfection of rice protoplasts were followed as described by Zhang et al. (2011) and images of cells with fluorescence were taken by confocal microscopy (LSM 510 META NLO DuoScan, Carl Zeiss).

### Scanning Electron Microscopy

To observe epidermal cells of lemma in rice grains by scanning electron microscopy (SEM), whole grains were coated with gold by the ion sputter machine (E-1010, Hitachi) and examined with SEM (Quanta 250, FEI, Hillsboro, OR, United States).

## Results

### *OsBDG1* May Act Downstream of *OsBUL1*

To identify the downstream genes of *OsBUL1* in the expressional hierarchy, comparison of transcriptomes of collars and panicles between WT and *osbul1* plants was conducted using rice 22k-oligo microarrays^1^. The *osbul1* is a mRNA null mutant caused by a T-DNA insertion in the first exon of *OsBUL1* gene (Jang et al., 2017).

**FIGURE 1:**
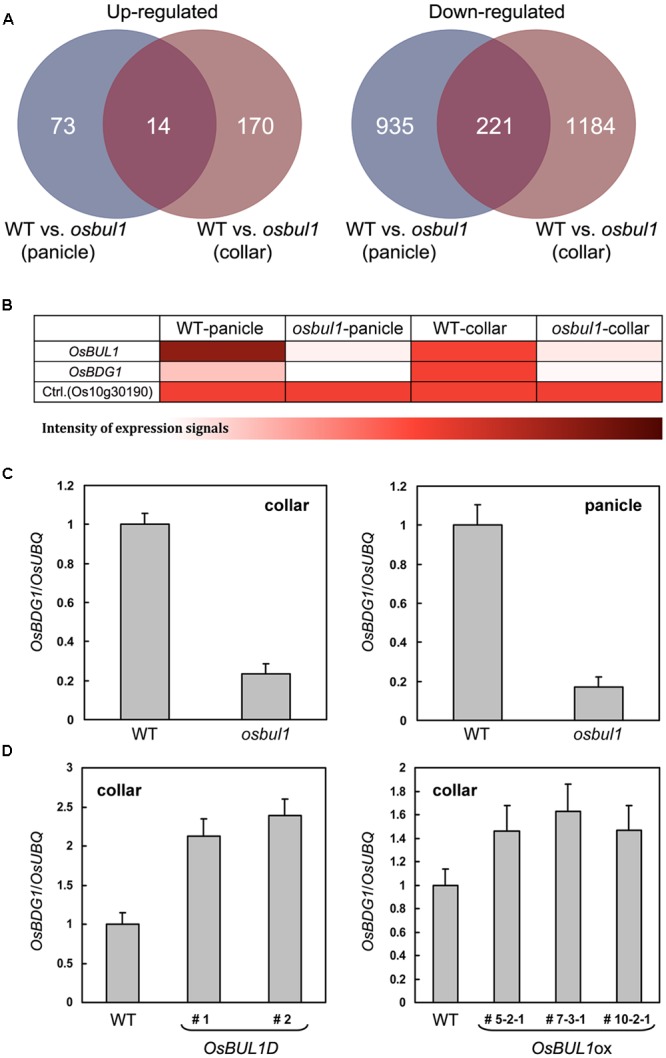
*OsBDG1* is a putative downstream gene of *OsBUL1* in expression. **(A)** Venn diagrams for upregulated and downregulated genes in the panicle and collar of *osbul1*. **(B)** A heat map based on microarray hybridization results shows that *OsBDG1* expression is reduced in *osbul1*. Color scale represents log signal values and Os10g30190 is a control showing similar signal values in the microarray hybridization between WT and *osbul1*. **(C)** Quantitative RT-PCR confirmed the reduced expression of *OsBDG1* in *osbul1* in collars and panicles. Conversely, expression level of *OsBDG1* is higher in an *OsBUL1* activation tagging line, *OsBUL1D* (Jang et al., 2017) and *OsBUL1* overexpressors (ox) than in the WT **(D)**. Data are the average of three or four independent experiments and normalized by *OsUBQ*. Error bars indicate SD.

**FIGURE 2:**
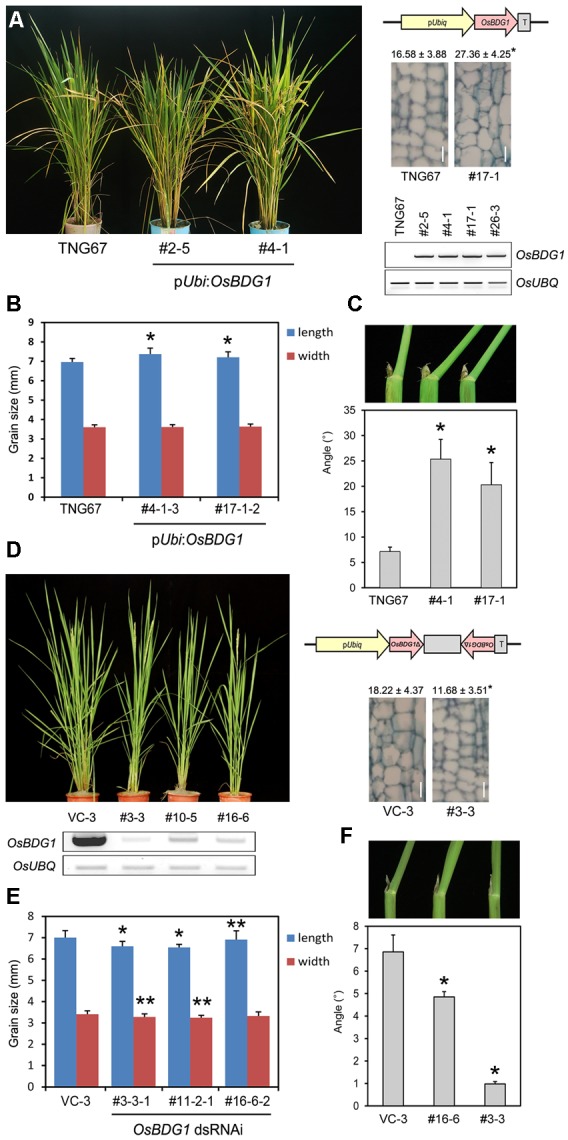
Transgenic rice plants with p*Ubi*:*OsBDG1* and *OsBDG1*-dsRNAi constructs. **(A–C)** Overexpression of *OsBDG1* driven by *ubiquitin* promoter caused increase of leaf angles with cell length in the lamina joint and grain size. Longitudinal sections of the second leaf lamina joint are shown in **(A)** with measured cell length. Values are means ± SD (μm, *n* > 20) as instructed by Zhang et al. (2009). (^∗^*P* < 0.01, Student’s *t*-test). Bar = 20 μm. Semi-quantitative RT-PCR was conducted for *OsBDG1* expression in transgenic rice plants together with WT. Twenty-four PCR cycles for both *OsBDG1* and *OsUBQ* were applied. Values of grain size and leaf angles are presented as means ± SD in **(B)** (*n* = 35; ^∗^*P* < 0.01, Student’s *t*-test) and **(C)** (*n* > 10; ^∗^*P* < 0.01, Student’s *t*-test), respectively. **(D–F)** Reduced expression of *OsBDG1* by dsRNAi-*OsBDG1* approaches resulted in erect leaves with shorter cells in lamina joint and smaller grains. The 384 bp-fragment of *OsBDG1* amplified by primers 5′ ATGGGGGCTCATTCTGCAGCGGCAGCTC 3′ and 5′ GTGCCACTCAGTGAATTCTTCTGAAGCTC 3′ was used for the dsRNAi construct. Semi-quantitative RT-PCR was conducted for *OsBDG1* expression in transgenic rice plants together with vector control (VC). Thirty-five and 24 PCR cycles were used for *OsBDG1* and *OsUBQ*, respectively. Longitudinal sections of the second leaf lamina joint are shown in **(D)** with measured cell length (μm, *n* > 20; ^∗^*P* < 0.01, Student’s *t*-test). Bar = 20 μm. Values of grain size and leaf angles are presented in **(E)** (*n* > 25; ^∗^*P* < 0.01; ^∗∗^*P* < 0.05, Student’s *t*-test) and **(F)** (*n* > 8; ^∗^*P* < 0.01, Student’s *t*-test), respectively.

Fourteen genes were upregulated, while 221 genes were downregulated in both collars and panicles of *osbul1* plants (**Figure 1A**). Among candidate genes, *OsBUL1 DOWNSTREAM GENE* (*OsBDG1*) was selected for further study since expression level of *OsBDG1* was reduced both in collars and panicles of *osbul1* plants (**Figure 1B**), and *OsBDG1* encodes a novel small protein containing a conserved LRR N-terminal domain (LRRNT) at the N-terminal part and three leucin-rich-repeat (LRR) domains toward its carboxyl terminus. In addition, its expression is known to be induced in transgenic rice plants overexpressing *Arabidopsis CYP90B1* which encodes for a sterol C-22 hydroxylase active in BR biosynthesis (Wu et al., 2008). To verify the microarray results, we performed expressional analyses on *OsBDG1* in collars and panicles of *osbul1* and WT plants, and found similar expression patterns to the microarray results (**Figure 1C**). On the contrary, the level of *OsBDG1* expression was higher in *OsBUL1*-overexpressing rice plants compared to the WT control (**Figure 1D**) indicating *OsBUL1* may act upstream of *OsBDG1*. Transgenic rice plants harboring p*Ubi*:*OsBDG1* show increased lamina inclination with elongated cells in the lamina joint and grain size (**Figures 2A–C**) whereas reduced expression of *OsBDG1* results in erect leaves with reduced cell length in lamina joint and smaller grains (**Figures 2D–F**). Phenotypes of overexpressors and dsRNAi lines for *OsBDG1* are similar to those of rice plants with increased *OsBUL1* expression and *osbul1*, respectively, supporting the expressional relationship between *OsBUL1* and *OsBDG1.*

### Identification of an Activation Tagging Line for *OsBDG1*

**FIGURE 3:**
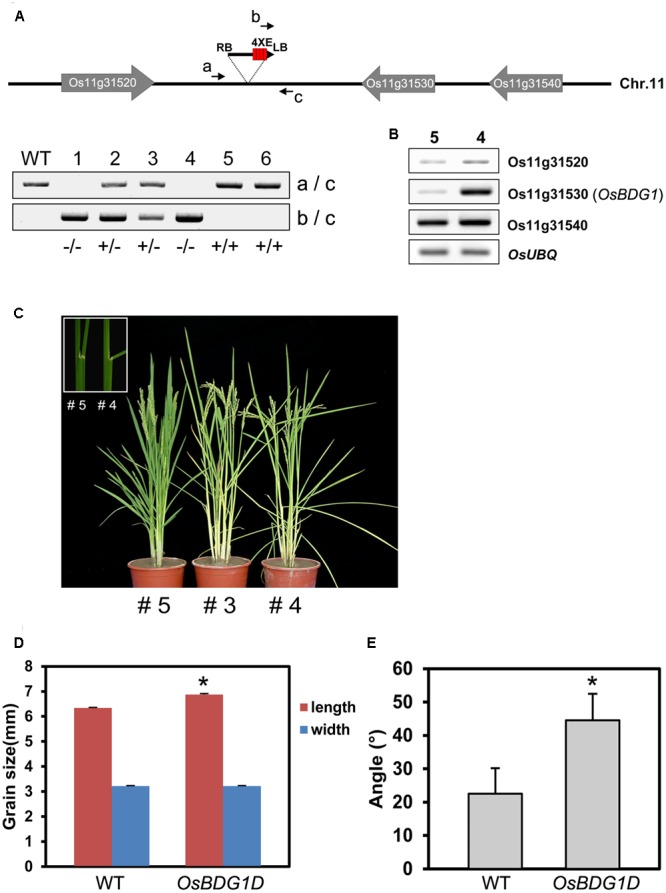
Identification and characterization of an *OsBDG1* activation tagging line. **(A)** T-DNA is inserted in the intergenic region between Os11g31520 and Os11g31530. Genotyping was conducted by primers, (a) 5′ CATAGGAACAGAAGGAGTAC 3′, (b) 5′ GACGAGAGTGTCGTGCTCCACCATG 3′ and (c) 5′ GAGGAGATTGTGGGCTCATG 3′. **(B)** Expression of genes located near the T-DNA insertion. **(C)** A gain-of-function line of *OsBDG1* showed increased lamina inclination. Lamina joint area of a WT segregant (#5; left) and a homozygous *OsBDG1D* line (#4) is shown in the box. **(D)** *OsBDG1D* plants produced grains with increased size and **(E)** caused increased leaf angles (the third leaf from the top of main stems). Error bars indicate SD. (^∗^*P* < 0.01, Student’s *t*-test).

A putative activation tagging line for *OsBDG1*, PFG_1B_05536 has been identified through the rice T-DNA database^2^ (An et al., 2003). Phenotypic analysis of the heterozygous population displayed a 3:1 ratio of *OsBDG1D* mutant phenotype to wild-type indicating *OsBDG1D* is a dominant mutant. T-DNA flanking sequence of the mutant was confirmed by PCR and comparison of the sequence through blastn search showed that the T-DNA was inserted in the 4.1 Kb downstream of Os11g31530 near Os11g31520 on chromosome 11 (**Figure 3A**). The expression of Os11g31530 was obviously influenced whereas the expression level of other genes located near the insertion position was not significantly affected (**Figure 3B**). The lamina angle increased more than two-fold in the activation tagging line with an increase of grain size (**Figures 3C–E**), which is similar to *OsBDG1* overexpressors.

### Expression of *OsBDG1* under the Control of *OsBUL1* Promoter

**FIGURE 4:**
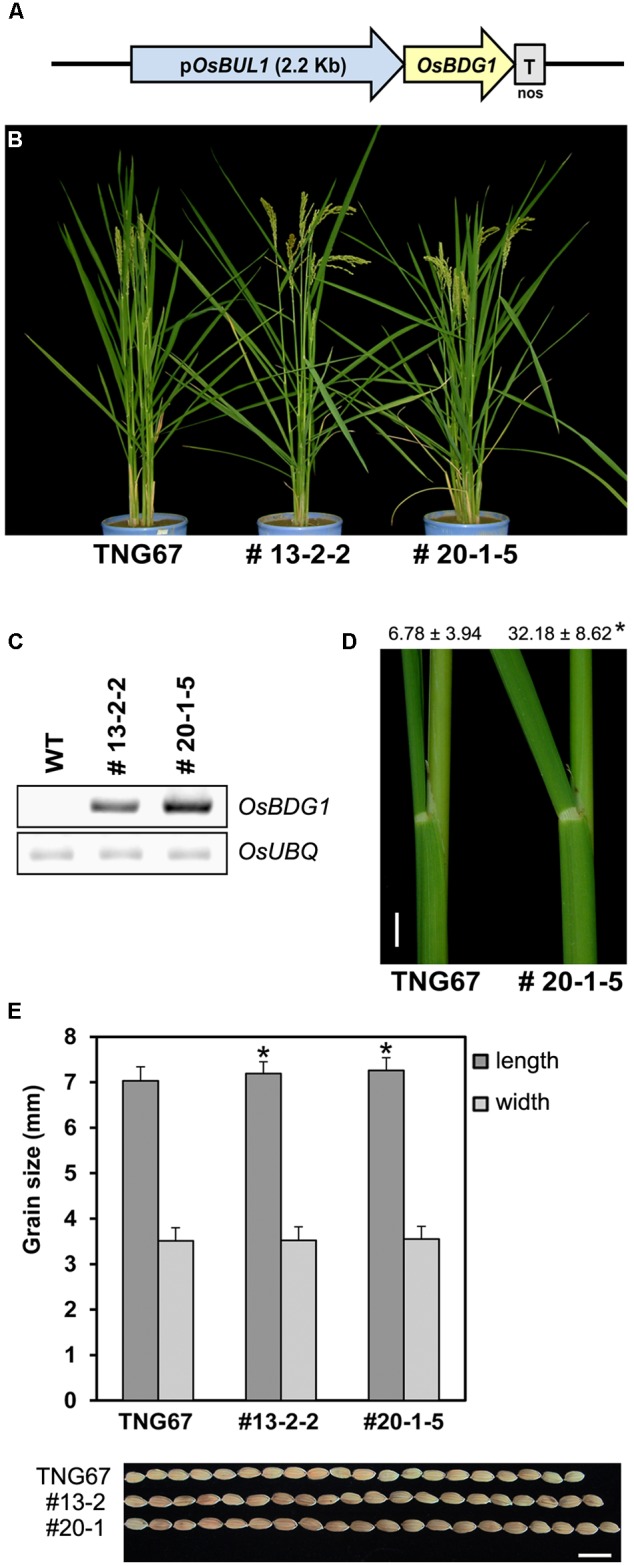
Phenotypic alterations of p*OsBUL1*:*OsBDG1* transgenic rice plants. **(A)** A simplified structure of p*OsBUL1*:*OsBDG1* construct. **(B)** Transgenic lines with p*OsBUL1*:*OsBDG1* construct exhibited conspicuous increase of leaf angles. **(C)** Expression of *OsBDG1* in collar between WT and transgenic plants. Semi-quantitative RT-PCR was conducted for *OsBDG1* expression in transgenic rice plants together with WT. Twenty-six PCR cycles for both *OsBDG1* and *OsUBQ* were applied. **(D)** Degree of leaf angles between WT and transgenic line, #20-1-5 at the second leaf from the top of the main stem. Values are given as means ± SD (degree; *n* > 14). (^∗^*P* < 0.01, Student’s *t*-test). Bar = 1 cm. **(E)** Transgenic lines produced grains with increased size. (^∗^*P* < 0.01, Student’s *t*-test). Bar = 1 cm.

To confirm the expression cascade of *OsBUL1* and *OsBDG1*, we made a construct for expression of *OsBDG1* under the control of the 2.2 kb-*OsBUL1* promoter (**Figure 4A**) preferentially active in lamina joints and panicles of rice (Jang et al., 2017). Transgenic rice containing the construct exhibited a dramatic increase in lamina inclination and grain size (**Figures 4B–E**), which was similar to the lamina inclination and grain phenotypes of the *OsBDG1* activation tagging line, PFG_1B_05536 and *OsBDG1*-overexpressing lines driven by *ubiquitin* promoter. Thus, expression of *OsBDG1* under the control of *OsBUL1* promoter phenocopies *OsBUL1* overexpression by *ubiquitin* promoter or activation tagging systems (Jang et al., 2017) suggesting that *OsBDG1* may act downstream of *OsBUL1*.

### *OsAP2* and *OsWRKY24* Are Upregulated by *OsBDG1* in Lamina Joints

**FIGURE 5:**
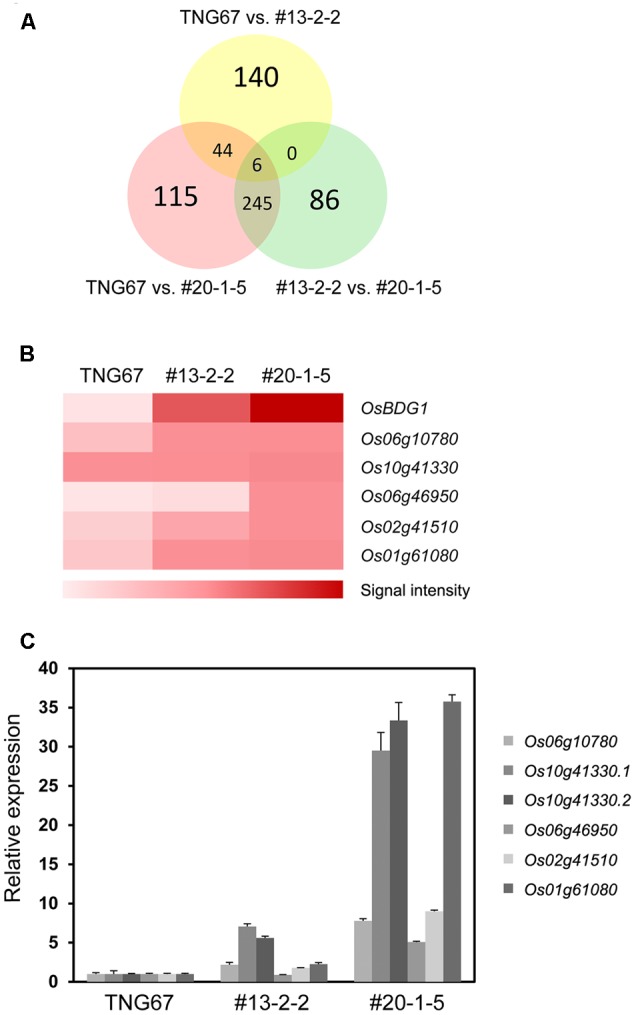
Identification of downstream genes of *OsBDG1* acting in the lamina joint. **(A)** A Venn diagram showing numbers of genes upregulated by *OsBDG1* in each comparison. Two independent T3 transgenic lines for p*OsBUL1*:*OsBDG1*, #13-2-2 and #20-1-5 were selected as mild and strong expressers of *OsBDG1* in collars, respectively, as shown in **Figure 4C**. **(B)** A heat map generated by microarray hybridization shows expression level of the five candidate genes identified as being gradually increased by *OsBDG1* expression. Color scale represents log signal values. **(C)** Verification of the expression level on each candidate gene in the collar of WT and transgenic lines, #13-2-2 and #20-1-5 by quantitative RT-PCR. Os10g41330 is known to produce two putative transcripts, Os10g41330.1 and Os10g41330.2 by alternative splicing (**Supplementary Figure S1**). Data are the average of three or four independent experiments and normalized by *OsUBQ*. Error bars indicate SD.

To identify candidate genes affected by *OsBDG1* in the lamina joint and also likely responsible for the increased lamina inclination, we compared transcriptomes of collars among two independent homozygous transgenic lines of p*OsBUL1*:*OsBDG1*, #13-2-2 and #20-1-5, and wild-type plants by microarray hybridization^3^. Since the expression level of *OsBDG1* in the collar of a #13-2-2 plant is lower than that of a #20-1-5 plant (**Figure 4C**), we selected genes that showed the same increasing or decreasing patterns in the order of WT, #13-2-2 and #20-1-5. Five genes were identified as being upregulated by *OsBDG1* in the lamina joint (**Figures 5A,B**) but no candidate was found to be downregulated in the comparison. Also, no significant difference in *OsBUL1* expression was detected among WT, #13-2-2 and #20-1-5 lines (GEO accession no. GSE93817 for microarray results). We confirmed the expression patterns of each gene by qRT-PCR (**Figure 5C**). The Os10g41330 (*OsAP2*) produced two transcripts, Os10g41330.1 and Os10g41330.2 by alternative splicing (**Supplementary Figure S1**) and both transcripts showed similar accumulation patterns. Based on the increased expression level of each gene in the transgenic plants, we focused on two genes, Os10g41330 (*OsAP2*) and Os01g61080 (*OsWRKY24*) encoding putative transcription factors containing an AP2 domain and WRKY domain, respectively. Conversely, in collars of *OsBDG1* dsRNAi lines, the expression of *OsAP2.2* and *OsWRKY24* is reduced without altered expression level of *OsBUL1* supporting the notion that the two genes are downstream of *OsBDG1* in expression (**Supplementary Figure S2**). However, the expression of *OsBUL1* and *OsBDG1* was positively affected by p*OsBUL1*:*OsAP2* and/or p*OsBUL1*:*OsWRKY24* (**Supplementary Figure S3**).

### Molecular Characterization of *OsBDG1, OsAP2*, and *OsWRKY24*

Spatiotemporal expression of *OsBDG1, OsAP2*, and *OsWRKY24* also overlapped in collars and growing panicles (**Supplementary Figure S4A**). Interestingly, the expression of the three genes was upregulated by phytohormones such as GA3 and BL, which affect cell elongation (**Supplementary Figure S4B**). Indeed, *OsBDG1* dsRNAi lines exhibited reduced, but transgenic rice containing p*OsBUL1*:*OsBDG1* showed increased sensitivity in BR response through lamina bending assays (**Supplementary Figure S4C**).

The OsBDG1 protein containing a short LRR motif was localized in the cytoplasm as well as the nucleus similar to OsBUL1 (Jang et al., 2017; **Supplementary Figure S5**). However, OsAP2.2 and OsWRKY24 proteins are localized in the nucleus and each protein shows a transcriptional activation activity in the yeast system (**Supplementary Figures S5, S6C**).

### Increased Lamina Inclination and Grain Size Phenotypes Are Observed in p*OsBUL1*:*OsAP2* and p*OsBUL1*:*OsWRKY24* Plants

**FIGURE 6:**
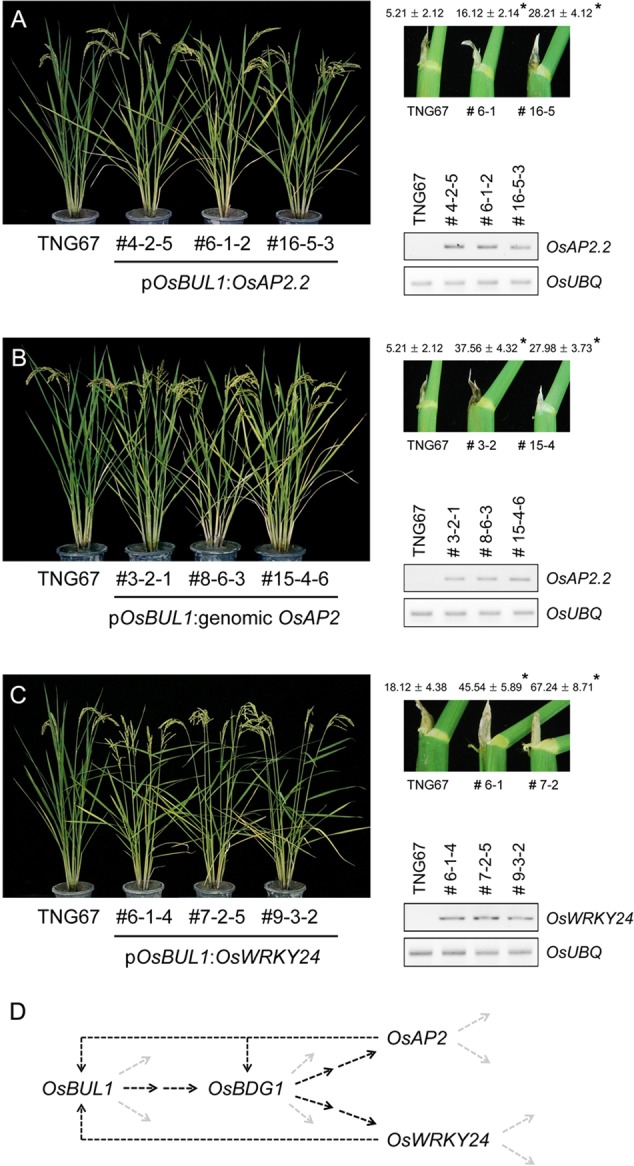
Generation of transgenic rice plants expressing *OsAP2* and *OsWRKY24* under the control of *OsBUL1* promoter. **(A,B)** Transgenic rice containing p*OsBUL1*:*OsAP2.2*, a short transcript of *OsAP2* (Os10g41330) or genomic *OsAP2* showed increased lamina inclination. The longer *OsAP2.1* transcript was not detected in p*OsBUL1*:genomic *OsAP2* plants. Leaf angle was measured with the second leaf from the top of the main stem. **(C)** Transgenic rice with p*OsBUL1*:*OsWRKY24* showed significant increase in leaf angles. The third leaf angles in the WT and transgenic lines were measured. RNAs were extracted from collars for cDNA synthesis and RT-PCR. Values for leaf angles are given as means ± SD (degree; *n* = 10 to 18). (^∗^*P* < 0.01, Student’s *t*-test). **(D)** Flow of genes acting in lamina inclination identified in this study. The dashed black lines were drawn based on results of expression analyses and transgenic approaches.

Transgenic rice plants containing p*OsBUL1*:*OsAP2.2*, a short transcript of Os10g41330 and p*OsBUL1*:genomic *OsAP2* were generated for phenotypic analyses of lamina angles and grain size (**Figure 6** and **Supplementary Figure S1**). Compared with the wild-type control, transgenic rice plants showed increased lamina inclination with increased amounts of *OsAP2.2* transcripts (**Figures 6A,B**). However, we could not detect the long form of the transcript, *OsAP2.1* in the transgenic plants containing p*OsBUL1*:genomic *OsAP2*. Transgenic rice for p*OsBUL1*:*OsWRKY24* also exhibited a significant increase in lamina angles (**Figure 6C**) indicating both *OsAP2.2* and *OsWRKY24* are likely affected by *OsBDG1* in the lamina joint. Of note, reduced expression level of both *OsAP2.2* and *OsWRKY24* was observed in panicles and lamina joints of *osbul1* plants (**Supplementary Figure S2**). Additionally, panicle morphology of transgenic rice was affected by the two transgenes: panicle branches were spread and grain size was also increased (**Supplementary Figure S3C**). Elongated epidermal cells of lemma were also observed with higher expression level of genes involved in cell elongation such as *OsExpansin* (*OsEXP*) genes, *OsXyloglucan endotransglucosylase/hydrolase1* (*OsXTH1*) and *Osxyloglucan endotransglycosylase related1* (*OsXTR1*) in spikelets of transgenic rice plants (**Supplementary Figure S6**; Cho and Kende, 1997; Uozu et al., 2000; Hara et al., 2014).

## Discussion

Controlling leaf angle and grain size of crop plants occupies a key position in the generation of elite lines with desirable agronomic traits in crop breeding programs. However, the mechanisms regulating the traits remain largely unknown. In this work, we identified putative genes acting downstream of *OsBUL1* for a positive effect on lamina inclination and grain size. First, with a view to investigating the downstream genes of *OsBUL1*, collection and comparison of transcriptomes of collars and panicles from WT and *osbul1* were conducted through microarray hybridization and, *OsBDG1* was selected as a putative downstream gene of *OsBUL1*. The expression of *OsBDG1* was reduced both in collars and panicles of *osbul1* and conversely increased in overexpressing plants and gain-of-function mutant of *OsBUL1*. Previously, *OsBDG1* was shown to be upregulated in transgenic rice ectopically expressing a sterol C-22 hydroxylase that controls BR levels in plants (Wu et al., 2008). Also, *OsBDG1* encodes a novel small protein with a rare structural feature; an LRR N-terminal domain (LRRNT) at the N-terminal part and three LRRs toward its carboxyl terminus. In this study, *OsBDG1* transcripts were shown to accumulate in response to BL and transgenic rice plants with overexpression and reduced expression of *OsBDG1* exhibited higher and lower sensitivities to BL, respectively, in lamina joint inclination bioassays (**Supplementary Figure S4**). Transgenic rice plants with increased expression of *OsBDG1* driven by *ubiquitin* promoter or *OsBUL1* promoter had phenotypes similar to those of rice plants including gain-of-function mutants of *OsBDG1, OsBUL1* and *OsBUL1* overexpressors (Jang et al., 2017) whereas *OsBDG1*-dsRNAi lines displayed similar phenotypes to *osbul1* in lamina inclination and grain size implying correlation between the expression and functional cascade between the two genes, although we cannot exclude the possibility of their having parallel genetic pathways.

Sequentially, two genes encoding nuclear proteins, *OsAP2* and *OsWARKY24* were identified as being downstream of *OsBDG1* in the lamina joint based on expression analyses of p*OsBUL1*:*OsBDG1* plants. Moreover, the expression level of the two genes was lower in *osbul1* as well as *OsBDG1* knockdown lines demonstrating an expression cascade among *OsBUL1, OsBDG1, OsAP2*, and *OsWRKY24* genes (**Supplementary Figures S2**). Recently, it was reported that *SMALL ORGAN SIZE1* (*SMOS1*) encoding an AP2-type transcriptional factor acts as an auxin-dependent regulator for cell expansion during organ size control (Aya et al., 2014) and SHOEBOX (SHB), another AP2/ERF transcription factor directly activates transcription of the GA biosynthesis gene *KS1* for the elongation of meristem cells in a developmental stage-specific manner (Li et al., 2015). Moreover, Wang et al. (2005) reported that OsWRKY11, a WRKY transcription factor, also regulates leaf inclination by analyzing a leaf angle mutant *large leaf angles* (*lla*), a T-DNA insertion mutant of *OsWRKY11*. Intriguingly, both *OsAP2.2* and *OsWARKY24* genes are upregulated by GA3 and slightly by BL and each protein exhibits transcriptional activation activity indicating that OsAP2.2 and OsWRKY24 may act as transcriptional activators to regulate the expression of downstream genes by phytohormones such as GA3 and/or BL. Functional characterization of the two genes by analyzing transgenic rice plants expressing each gene in the place where *OsBUL1* is expressed also suggests that they influence cell elongation in a positive manner. Thus, we found expressional and putative functional relationships of genes involved in the promotion of cell elongation for increased lamina inclination and grain size of rice although it remains unknown whether direct regulation is available among these genes (**Figure 6D**). It would be of value to examine transcripts affected by the two transcription factors, OsAP2.2 and OsWRKY24 in the lamina joint of rice. The next challenge is to produce rice plants with erect leaves and larger grain size. A trial for reduced expression of genes such as *OsBUL1* and/or *OsBDG1* under the control of *OsBC1* promoter (Jang et al., 2017) is worth conducting to test whether rice plants can exhibit an erect leaf trait without compromising on the grain size.

## Conclusion

We have identified a series of novel rice genes related to increased lamina inclination and grain size based on exploitation of their expressional relationships and evaluated their functional roles by molecular genetic approaches. These results indicate that they are good candidates that may be further studied or used together with proper promoters for improving crop productivity through desirable plant architecture in the future.

## Accession Numbers

Genes in this article can be found in the GenBank/EMBL or RiceGE databases under the following accession numbers: *OsAP2* (Os10g41330), *OsBDG1* (Os11g31530), *OsBUL1* (Os02g51320), *OsEXPA1* (Os04g15840), *OsEXPA2* (Os01g60770), *OsEXPA3* (Os05g19570), *OsEXPA4* (Os05g39990), *OsWRKY24* (Os01g61080), *OsXTH1* (Os04g51460), *OsXTR1* (Os11g33270). GEO accession number for microarray data in this study is GSE93817.

## Author Contributions

SJ designed the experiments. SJ and H-YL performed the experiments, analyzed the data. SJ wrote the article.

## Conflict of Interest Statement

The authors declare that the research was conducted in the absence of any commercial or financial relationships that could be construed as a potential conflict of interest.

## Footnotes

1 http://www.phalanxbiotech.com

2 http://signal.salk.edu/cgi-bin/RiceGE

3 http://www.phalanxbiotech.com

## Supplementary Material

FIGURE S1 Sequence of *OsAP2* genomic clone used for construction of p*OsBUL1*:genomic *OsAP2*. The long transcript of *OsAP2* (*OsAP2.1*) is marked with uppercase and the yellow block is for *OsAP2.2*, a short transcript. Introns are shown in lowercase. Underlined sequences are quantitative PCR primers for *OsAP2.1* and double underlined sequences are for *OsAP2.2*. Start and stop codons are presented with red color.

FIGURE S2 Expression of *OsAP2* and *OsWRKY24* is reduced in *osbul1* plants. **(A)** A heat map showing that the expression level of *OsAP2* and *OsWRKY24* as well as *OsBDG1* is reduced in *osbul1* plants. Color scale represents log signal values. **(B,C)** Experimental confirmation of the expression level of *OsAP2.2* and *OsWRKY24* compared with microarray data in **(A)**. The expression of *OsAP2.2* and *OsWRKY24* is reduced in collars **(D)** and panicles **(E)** of *OsBDG1*-dsRNAi lines without significant alteration of *OsBUL1* expression **(F)**. Data are the average of two or three independent experiments and normalized by *OsUBQ*. Error bars indicate SD.

FIGURE S3 Expression of *OsBUL1* and *OsBDG1* is affected by p*OsBUL1*:*OsWRKY24* and/or p*OsBUL1*:*OsAP2*. **(A)** *OsBUL1* expression is increased by p*OsBUL1*:*OsWRKY24* and p*OsBUL1*:*OsAP2.2* in panicles while *OsBDG1* transcripts **(B)** are accumulated only in p*OsBUL1*:*OsAP2.2* plants. Data are the average of three independent experiments and normalized by *OsUBQ*. Error bars indicate SD. **(C)** Panicle branches exhibit increased angles in p*OsBUL1*:*OsAP2.2* and p*OsBUL1*:*OsWRKY24* transgenic rice plants. Arrowheads indicate nodes for panicles. Bar = 5 cm. Length of grains from the transgenic lines also show a significant increase. (mm; *n* > 25). (^∗^*P* < 0.01, Student’s *t-*test).

FIGURE S4 Expression pattern of *OsBDG1, OsAP2.2* and *OsWRKY24* analyzed by quantitative RT-PCR and lamina inclination of p*OsBUL1*:*OsBDG1* and *OsBDG1*-dsRNAi plants in lamina bending assays. **(A)** Spatiotemporal expression patterns of genes studied. Error bars indicate SD of three technical repeats. **(B)** Expression analyses of genes with treatment of GA3 and BL at 24 h time points after treatment. Data are the average of three or four independent experiments and normalized by *OsUBQ* or *OsAct*. Error bars indicate SD of three biological replicates. Differences between the mock and hormone treated samples are highlighted with ^∗^*P* < 0.01; ^∗∗^*P* < 0.05 with Student’s *t-*test. **(C)** Lamina joint inclination bioassays with various concentrations of BL.

FIGURE S5 Subcellular localization of proteins. **(A)** YFP:OsBDG1 and CFP:OsBC1 were co-transformed into rice protoplasts. OsBDG1 is localized in the cytoplasm as well as the nucleus while OsBC1 (Jang et al., 2017) is only found in the nucleus. **(B)** CFP:OsBDG1 and YFP:OsBUL1 were co-transformed into rice protoplasts. OsBDG1 and OsBUL1 are co-localized in the cell. **(C,D)** CFP:OsAP2.2 and CFP:OsWRKY24 are localized in the nucleus. Bar = 5 μm.

FIGURE S6 Morphological alteration of epidermal cells of lemma from p*OsBUL1*:*OsAP2.2* and p*OsBUL1*:*OsWRKY24* and expression pattern of genes involved in cell elongation. **(A)** Elongated epidermal cells of lemma from p*OsBUL1*:*OsAP2.2* and p*OsBUL1*:*OsWRKY24* transgenic plants compared to those of WT. Bar = 100 μm. **(B)** Expression of genes involved in cell elongation in spikelets of transgenic lines. Data are the average of three independent experiments and normalized by *OsAct*. Error bars indicate SD. **(C)** Transcriptional activation activity analyses of OsAP2.2 and OsWRKY24. Unlike BD-OsBDG1, both BD-OsAP2.2 and BD-OsWARK24 fusion proteins displayed a transcriptional activation activity in the yeast system described by Jang et al. (2015).

FIGURE S7 Primers used for expression analyses in this study.
