# Supplementary figures and images for: Oryza sativa BRASSINOSTEROID UPREGULATED1 LIKE1 Induces the Expression of a Gene Encoding a Small Leucine-Rich-Repeat Protein to Positively Regulate Lamina Inclination and Grain Size in Rice
Source: Front Plant Sci. 2017 Jul 17;8:1253. doi: 10.3389/fpls.2017.01253 (PMC5511847; doi:10.3389/fpls.2017.01253)

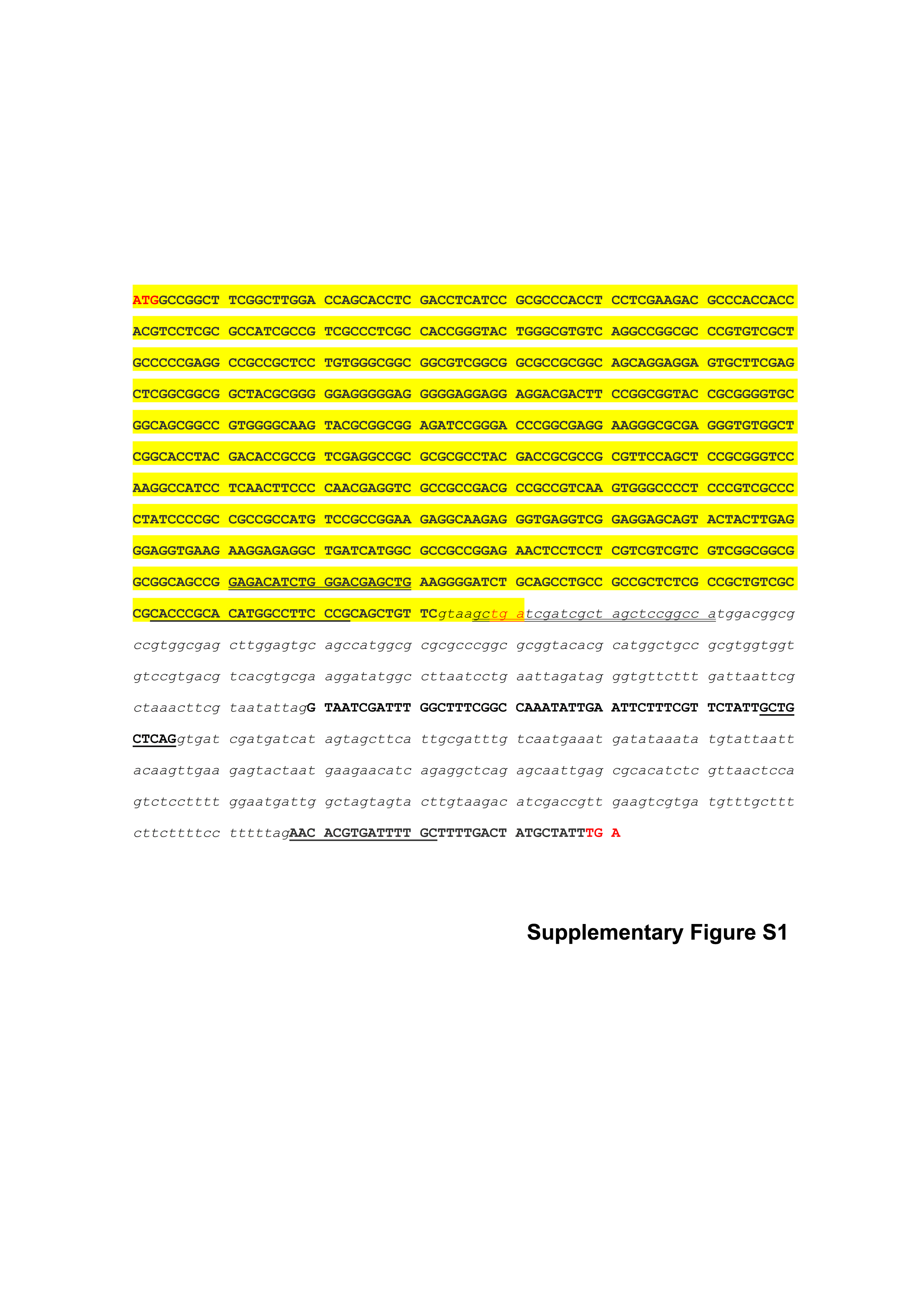

Supplement: FIGURE S1 — Sequence of OsAP2 genomic clone used for construction of pOsBUL1:genomic OsAP2. The long transcript of OsAP2 (OsAP2.1) is marked with uppercase and the yellow block is for OsAP2.2, a short transcript. Introns are shown in lowercase. Underlined sequences are quantitative PCR primers for OsAP2.1 and double underlined sequences are for OsAP2.2. Start and stop codons are presented with red color. [file Image_1.JPEG]

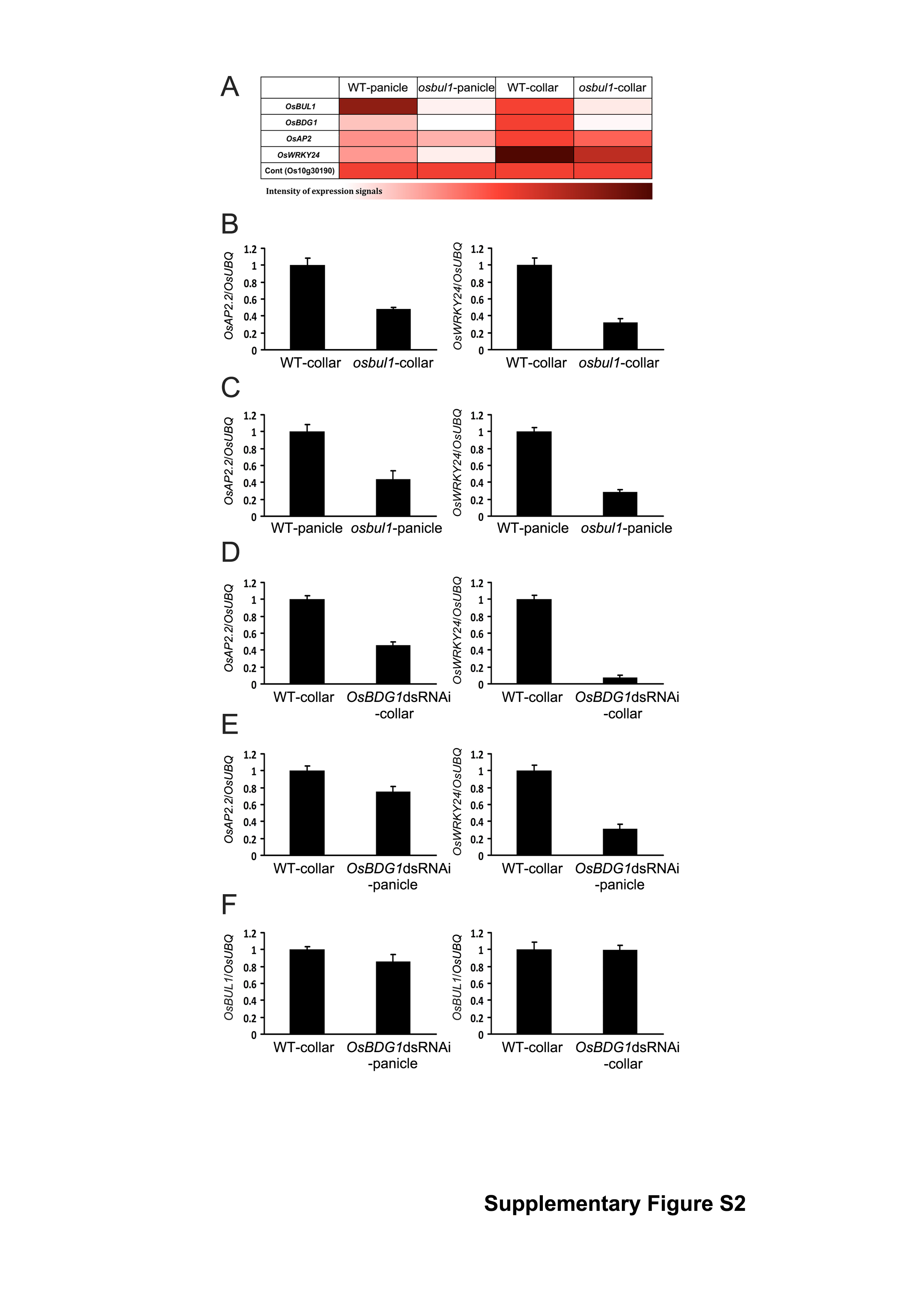

Supplement: FIGURE S2 — Expression of OsAP2 and OsWRKY24 is reduced in osbul1 plants. (A) A heat map showing that the expression level of OsAP2 and OsWRKY24 as well as OsBDG1 is reduced in osbul1 plants. Color scale represents log signal values. (B,C) Experimental confirmation of the expression level of OsAP2.2 and OsWRKY24 compared with microarray data in (A). The expression of OsAP2.2 and OsWRKY24 is reduced in collars (D) and panicles (E) of OsBDG1-dsRNAi lines without significant alteration of OsBUL1 expression (F). Data are the average of two or three independent experiments and normalized by OsUBQ. Error bars indicate SD. [file Image_2.JPEG]

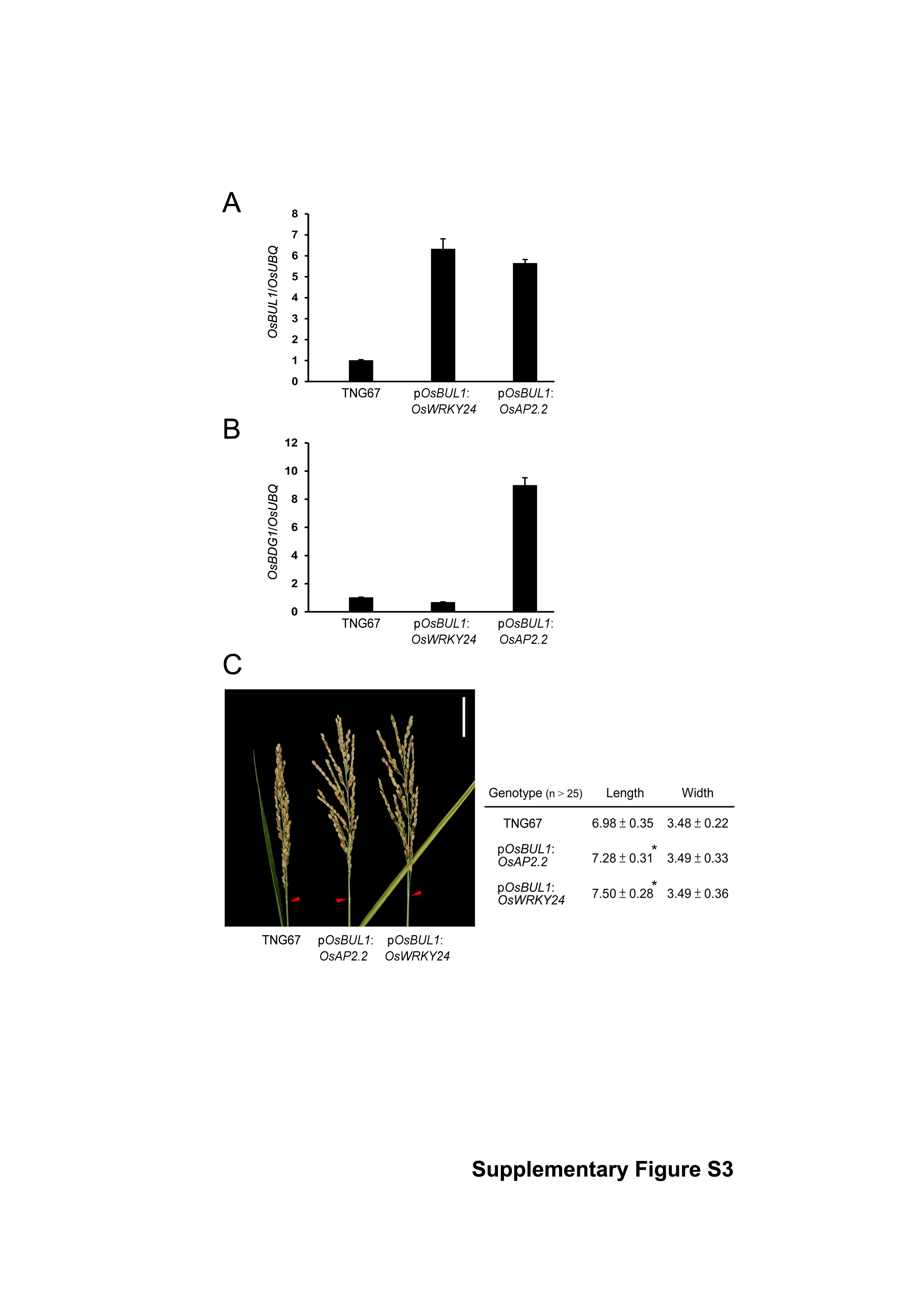

Supplement: FIGURE S3 — Expression of OsBUL1 and OsBDG1 is affected by pOsBUL1:OsWRKY24 and/or pOsBUL1:OsAP2. (A) OsBUL1 expression is increased by pOsBUL1:OsWRKY24 and pOsBUL1:OsAP2.2 in panicles while OsBDG1 transcripts (B) are accumulated only in pOsBUL1:OsAP2.2 plants. Data are the average of three independent experiments and normalized by OsUBQ. Error bars indicate SD. (C) Panicle branches exhibit increased angles in pOsBUL1:OsAP2.2 and pOsBUL1:OsWRKY24 transgenic rice plants. Arrowheads indicate nodes for panicles. Bar = 5 cm. Length of grains from the transgenic lines also show a significant increase. (mm; n > 25). (∗P < 0.01, Student’s t-test). [file Image_3.JPEG]

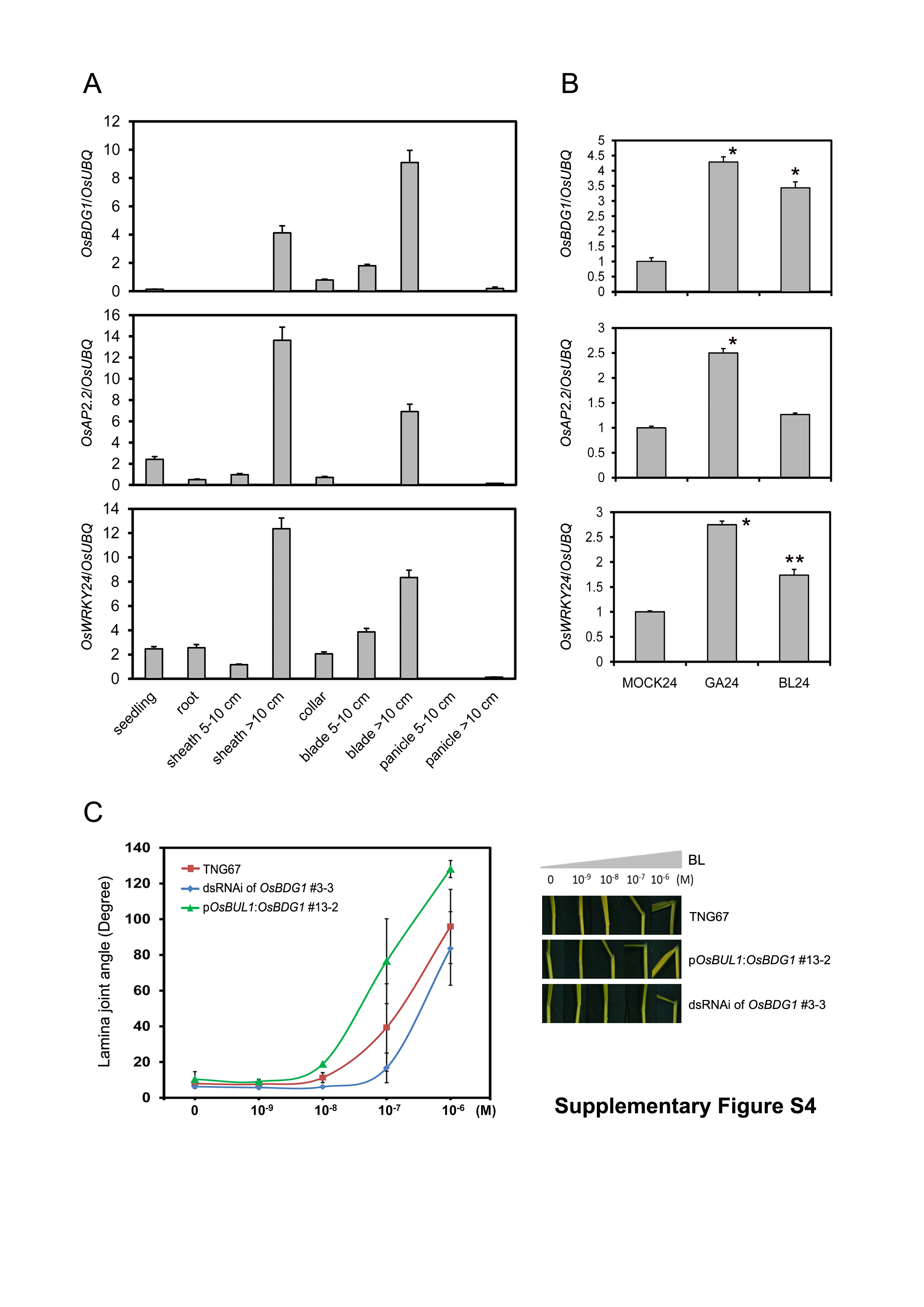

Supplement: FIGURE S4 — Expression pattern of OsBDG1, OsAP2.2 and OsWRKY24 analyzed by quantitative RT-PCR and lamina inclination of pOsBUL1:OsBDG1 and OsBDG1-dsRNAi plants in lamina bending assays. (A) Spatiotemporal expression patterns of genes studied. Error bars indicate SD of three technical repeats. (B) Expression analyses of genes with treatment of GA3 and BL at 24 h time points after treatment. Data are the average of three or four independent experiments and normalized by OsUBQ or OsAct. Error bars indicate SD of three biological replicates. Differences between the mock and hormone treated samples are highlighted with ∗P < 0.01; ∗∗P < 0.05 with Student’s t-test. (C) Lamina joint inclination bioassays with various concentrations of BL. [file Image_4.JPEG]

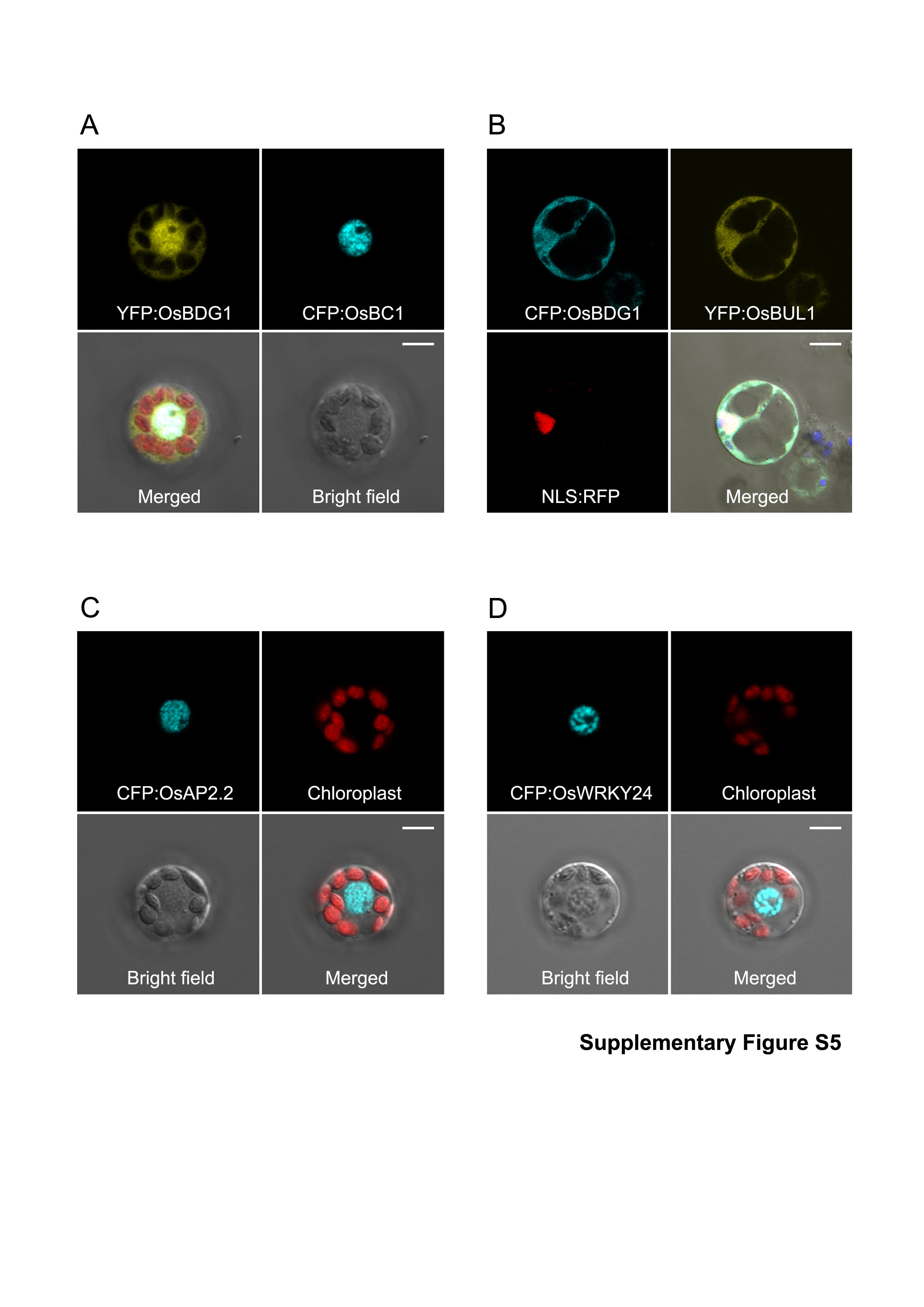

Supplement: FIGURE S5 — Subcellular localization of proteins. (A) YFP:OsBDG1 and CFP:OsBC1 were co-transformed into rice protoplasts. OsBDG1 is localized in the cytoplasm as well as the nucleus while OsBC1 (Jang et al., 2017) is only found in the nucleus. (B) CFP:OsBDG1 and YFP:OsBUL1 were co-transformed into rice protoplasts. OsBDG1 and OsBUL1 are co-localized in the cell. (C,D) CFP:OsAP2.2 and CFP:OsWRKY24 are localized in the nucleus. Bar = 5 μm. [file Image_5.JPEG]

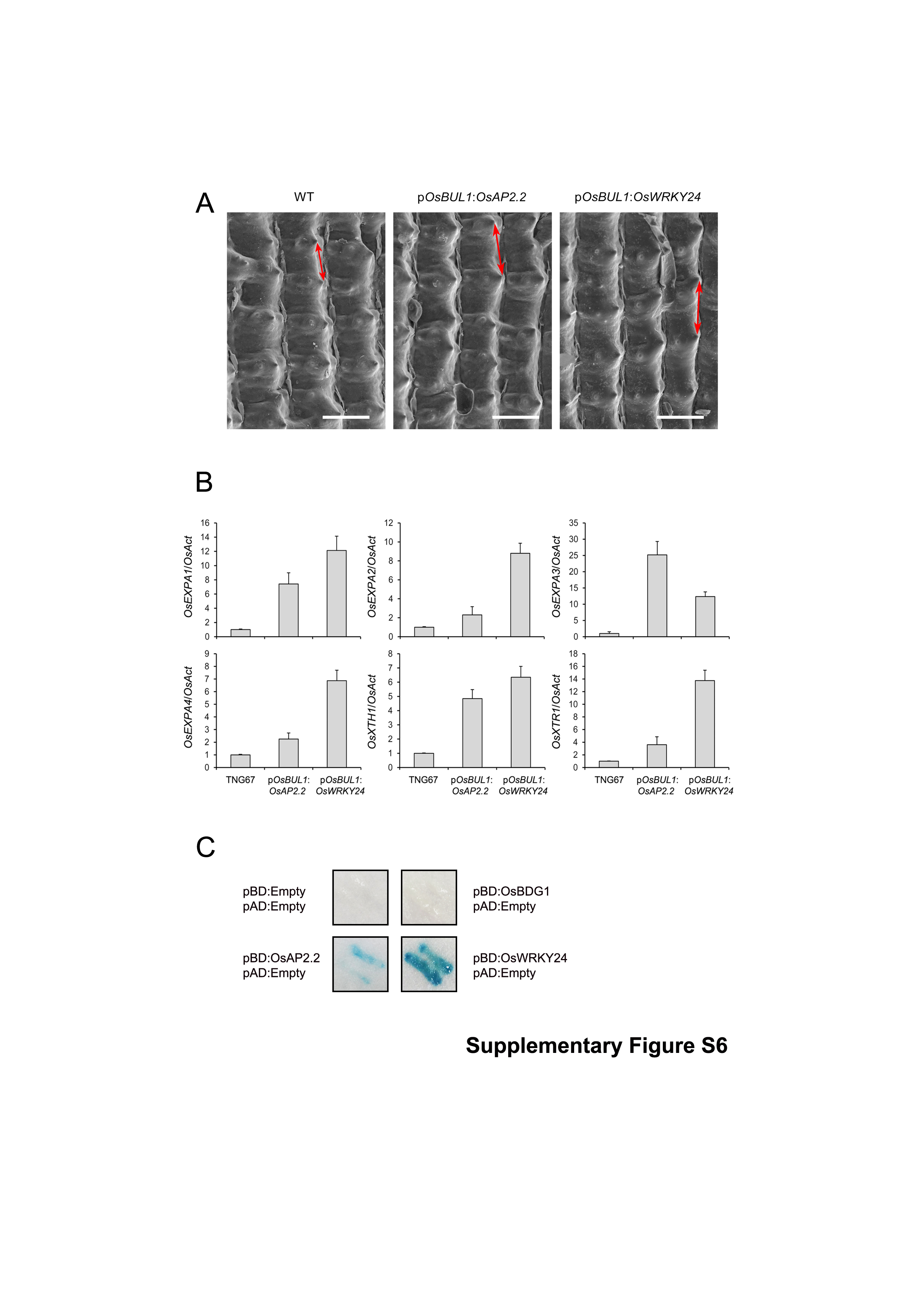

Supplement: FIGURE S6 — Morphological alteration of epidermal cells of lemma from pOsBUL1:OsAP2.2 and pOsBUL1:OsWRKY24 and expression pattern of genes involved in cell elongation. (A) Elongated epidermal cells of lemma from pOsBUL1:OsAP2.2 and pOsBUL1:OsWRKY24 transgenic plants compared to those of WT. Bar = 100 μm. (B) Expression of genes involved in cell elongation in spikelets of transgenic lines. Data are the average of three independent experiments and normalized by OsAct. Error bars indicate SD. (C) Transcriptional activation activity analyses of OsAP2.2 and OsWRKY24. Unlike BD-OsBDG1, both BD-OsAP2.2 and BD-OsWARK24 fusion proteins displayed a transcriptional activation activity in the yeast system described by Jang et al. (2015). [file Image_6.JPEG]
